# Interview: a conversation with Vishva M Dixit on his journey from remote African village to apoptosis, necroptosis and the inflammasome

**DOI:** 10.1038/s41418-019-0294-9

**Published:** 2019-02-08

**Authors:** Vishva M. Dixit

**Affiliations:** Genentech, Inc., South San Francisco, CA United States

## Abstract

Vishva M. Dixit, M.D., Vice President of Physiological Chemistry at Genentech, Inc. has made many contributions to biomedicine, and his early work on apoptosis is prominent in introductory textbooks of biology and medicine.

He is a member of the National Academy of Sciences, the National Academy of Medicine, the American Academy of Arts and Sciences, and a Foreign Member, European Molecular Biology Organization.

Additionally, he serves on the Boards of the Gates Foundation, Howard Hughes Medical Institute, and Keystone Symposia.

CDD: Tell us about your early years. Where did you grow up?

I was born in Kenya, East Africa (Fig. 1), in the small town of Kisii. At that time—the mid-1950s—Kenya was still a British colony and we were all subjects of the Queen. It was quite surreal with a sharp demarcation of races, each with their own schools, hospitals, and civic centers. My parents were both physicians and as part of the colonial service in the 1940s, were sent to Kenya from India. Initially, they were posted to the “Northern Frontier District,” a forbidding place close to the border of Somalia where, according to them, the scorching sun, sand teeming with poisonous scorpions, and murderous *shifta* (bandits) reigned supreme. For reasons lost in history, the British had built a number of prison camps in this most inhospitable of places and the associated clinic was run by my parents.

**Fig. 1:**
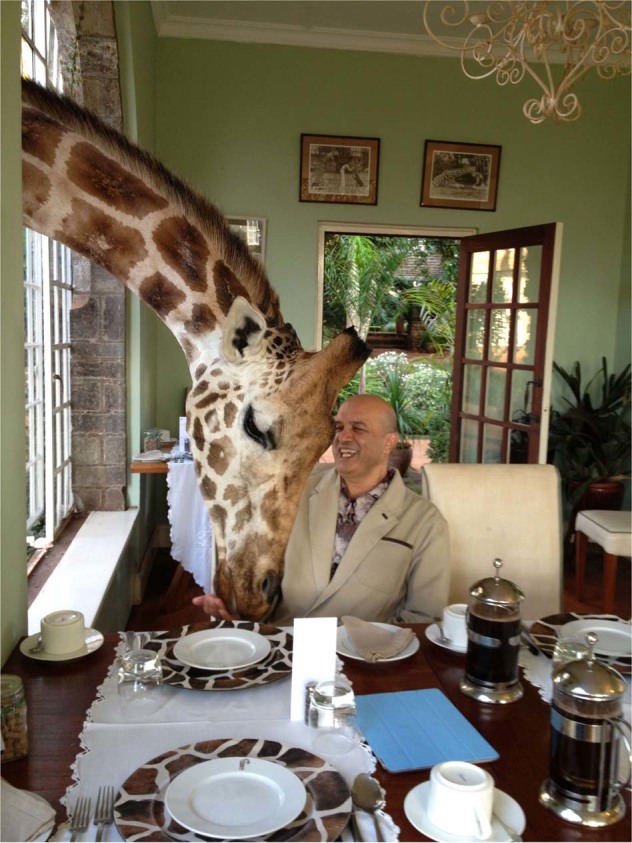
Ambushed by a giraffe (!) on a recent vacation in Kenya

After many of the camps disbanded, my mother soon left to seek safety and solace in Nairobi, the big city and capital of Kenya. My father, however, soldered on, and in later years, would regale us with his stories. One still comes to mind: his orderly awakened him one night, as there was an emergency. A Somali man wandering the scrubland had been attacked by a hyena which had clamped its jaws over the man’s forearm. Wielding a machete with his free hand, the man cut off the head of the hyena–which was still attached to his arm! The hyena has the strongest jaws in the animal kingdom, so little wonder it took my father the better part of the night dissecting it off the man’s mangled arm.

After leaving the colonial service, my parents set up their private practice in a number of small towns, eventually settling in Kericho, where I grew up and attended primary school (Fig. 2). Kericho was, and still is, the center of the tea industry. Nestled in the Kenyan Highlands at 7000 feet above sea level, Kericho’s hilly countryside was a sea of emerald green from the densely planted tea bushes. After independence in 1963 and the end of apartheid-like policies, I attended a “European” school that had been desegregated. Understandably, there was initial animosity to those that looked different, but that was soon forgotten as we became well acquainted.

**Fig. 2:**
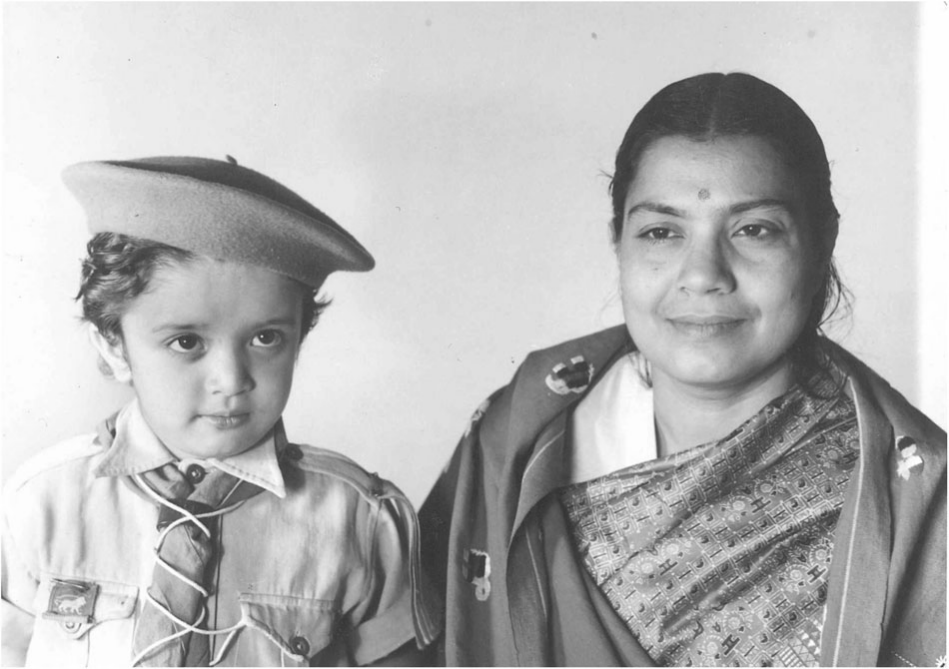
Early childhood: as a boy scout with Mother

CDD: What got you interested in Science?

My parents were often busy in the evenings with house calls and to keep me occupied, they bought a series of science books from Time-Life publishers that instilled the excitement of discovery in me. As a 10-year old, I was fascinated by the prospect that one could still be an explorer—a discoverer of new worlds using the tools of science. Moreover, it seemed that one could even achieve immortality through such discoveries. Heartened by this admittedly naive introduction to the joys of becoming a science explorer, I decided on my career.

CDD: How did you end up choosing Medicine?

To accommodate my attending a decent high school and my brother’s medical school, we moved to the capital city, Nairobi. I was an average student and muddled through high school with the turbulence of adolescence inflicting more than its usual trauma. Not unusual for conservative immigrant Indian families of that time, a parental career decision was imposed upon me: come hell or high water, I was to pursue medicine like my older sister and brother. So much for the angst in making a career decision—it was done for me!

CDD: What was Medical School like?

I went to medical school in Nairobi and it was a revelation on so many levels. The teaching in many subjects was outstanding. In particular, physiology taught by an erudite Sri Lankan, Professor Hettiaratchi, revealed the foundational logic to medical science: the constancy of the inner milieu as specified by the great French physiologist, Claude Bernard, and evolutionary adaptation to environmental pressures as described by the greatest of all biologists, Charles Darwin.

I was also exposed to terribly sick patients. As people had limited means, they came to the hospital only when the disease was well advanced. Most tragic were cases of childhood dehydration due to acute diarrhea—in the late 1970s, it was responsible for the tragic deaths of an astounding five million children each year, a number truly hard to comprehend! Dehydration at that time was treated with intravenous replenishment of fluids, but this required access to a clinic or hospital that could be miles away and difficult to access due to inadequate roads and transportation. Not surprisingly, by the time they reached us, mortality was high in the best of circumstances.

In the 1980s, however, the miracle of oral rehydration therapy was introduced and children could be rapidly treated in the countryside by someone with absolutely no medical training. Indeed, the prestigious medical journal, *The Lancet*, described oral rehydration therapy as “potentially the most important medical advance of the last century.”

CDD: Given this, did you consider going into Public Health?

It dawned on me that to have a meaningful impact in a country like Kenya, where the infant mortality rate was 120 per 1000 and the health budget a few dollars per person per year, public health measures, namely, clean water and vaccinations, had to be the very top priorities by a wide margin. These, however, are policy and political decisions that are ensnared in the economic fortunes of a country and most vitally, proper governance. This realization disillusioned my interest in pursuing public health, as I felt there was little I could do to influence politics.

CDD: Any other memories from Medical School?

Towards the end of my clinical training in the late 1970s, I worked in the tropical medicine unit run by Philip Rees, an extraordinary physician. We began to see famished, skeletal-looking patients who had been referred to our unit to work up “fever of unknown origin.” All the tests turned out negative. We sent samples to the Royal Tropical Institute in the Netherlands and the London Institute of Tropical Medicine, but to no avail. The analyses were uniformly negative and the patients rapidly succumbed to a mysterious wasting illness.

Years later, I came to realize that these patients must have represented the beginning of the AIDS epidemic—they were mostly long-haul truck drivers who serviced the route from the coastal city of Mombasa to Rwanda and the Congo. They were undoubtedly exposed to the virus from unprotected sex. This was my brush with an early epidemic and it underscored the helplessness of ignorance. All we could do was watch their rapid decline. Clearly there was a desperate need for new knowledge in medicine.

CDD: How did you end up coming to the United States?

My childhood fascination with discovery had never faltered and I began to seriously consider a career in basic medical research. This meant going overseas, given the lack of resources and funding in Kenya. Since my sister and brother had immigrated to the United States, that was a natural place to consider. But I had a medical degree from Nairobi, hardly the hotbed of biomedical research. I had no bench research experience and it was only through the good graces of Dr. Hettiaratchi that I had dabbled in some clinical research. We published a study on the rural predisposition of subacute sclerosing panencephalitis in *Developmental Medicine and Child Neurology* [1]. Additionally, I somehow convinced an editor at *The Lancet*, who must have been befuddled to receive a poorly typed manuscript from a medical student in the middle of Africa, to publish a hypothesis on the possible biochemical cause for clinical depression in scurvy [2].

At least I had a couple of publications under my belt, but first and foremost, I had to get my medical degree recognized in the U.S. by taking a number of examinations that went by various acronyms—ECFMG, VQE, FLEX—inflicting varying degrees of terror in the hearts of foreign medical graduates. Fortunately, the rigorous training I had received in Nairobi made these exams a total breeze, but I was still left with the problem of gaining admission to a residency program that would allow me to undertake research.

In this regard, I owe my brother tons, as he was instrumental in helping me secure a residency slot in the storied Department of Pathology at Washington University in St. Louis. Through a combination of determination and hard work, he obtained a coveted residency position in the Internal Medicine program at Washington University, despite being a foreign medical graduate. Once ensconced, he convinced Jay McDonald, the head of the Laboratory Medicine program, to give me a break, changing the trajectory of my career.

CDD: Tell us about your residency training?

Coming from Kenyatta National Hospital—a crowded 3000-bed hospital with suffering sick people piled in the corridor and two patients to a bed at times—to the pristine, efficient mecca of Barnes Hospital in St. Louis, was a change of staggering proportions. The enormous, architecturally stunning hospital was conspicuously clean, carpeted, and practically deserted!

The first year of clinical training in laboratory medicine I rotated through various functions like blood banking and microbiology that make up the discipline. On the first day, I made quite an impression on the departmental secretary, when in search of an eraser, I asked her for a rubber! With raised eyebrows, she set me right in no uncertain terms. Things didn’t get much better when later in the week, I mentioned that I needed time off to join AA—which in my mind stood for the Automobile Association, but in hers, it confirmed her worst fears—Alcoholics Anonymous.

The training was nothing short of a revelation. We were encouraged to be inquisitive and question published work. This was brought to a fore at weekly presentation meetings—known amongst the residents as the “shark tank.” During this session, one literally had to run a gauntlet of probing questions that at times bordered on impolite—and that is putting it mildly! This, however, was the absolute best training I received, as it totally altered my perception of what it meant to be a scientist. One had to question perceived wisdom in a most thoughtful, precise, and rigorous manner and draw one’s own conclusions.

CDD: What was your initial exposure to research like?

The Pathology program was incredibly enlightened, as after the first clinical year, it strongly encouraged residents to train in bench research. Most of my co-trainees were MD/PhDs who had already published papers, so they had no difficulty in finding a research position in the top laboratories. If anything, they were highly sought after. I, unfortunately, had no such credentials, but the department came to my aid by promising to continue to pay my salary, so I’d essentially be a free pair of hands for any laboratory that would accept me.

I was extremely fortunate to end up with William Frazier, who was a professor in the famed biochemistry department, former home of Carl and Gerty Cori and other Nobel laureates. I remember the interview like it was yesterday. Bill Frazier asked me if I’d ever used a Pipetman—to which I responded in the negative. Ever used a pH meter—again I had to disappoint him. I thought my chances were zero to none that he’d take such a naïve ignoramus, one who had absolutely no laboratory experience.

To my absolute delight, he gave me a position. Since he worked at the bench doing his own experiments, he asked me to be his apprentice. So I’d tag along, having the good fortune of a professor showing me how to make buffers, pour gels, and work with radioactive tracers and culture cells.

Conceived with Sam Santoro, a young professor in Pathology, the project had to do with platelet aggregation—specifically, the role of thrombospondin, a large extracellular protein, in gluing platelets together and forming subsequent blood clots. The project involved isolating the protein from recently expired human platelets from the blood bank.

Since expiration could happen at any time and the protein had to be isolated immediately, I spent many a night in a cold room struggling with column purification of an intrinsically unstable protein that tended to ruinously aggregate if one stopped at any stage of the process. In this endeavor, I was greatly aided by Karen O’Rourke, a lab technician who, as fate would have it, still works with me 35 years later!

It took a couple of years to master efficient and reproducible purification after which the project took off. Its eventual cloning was a collaboration with Peter Rotwein, a molecular endocrinologist who possessed mastery over what at the time was a mysterious art: genetic engineering.

CDD: Did you get to publish or present your work on Thrombospondin?

Yes—the work resulted in a number of publications and my first oral presentation at an international meeting, the American Society of Cell Biology. I was a nervous wreck, but the talk went fine. What struck me, however, was the massive size of the research enterprise in cell and molecular biology. It wasn’t just a few dozen attendees, but a few thousand! In addition, the talks covered just about every aspect of biology conceivable! Disconcertingly, there were literally acres of scientific posters—one would need a lifetime just to wade through them.

I found this to be somewhat terrifying—how could one make a visible contribution in a field crowded by so many talented researchers?! Was my fate going to be to dot the i’s and cross the t’s, or would I be able to make a worthy contribution? I felt like a microscopic cog in a very large machine and was overcome with a sense of dread and inadequacy that, to varying extents, haunted me through much of my career.

CDD: Tell us about your first real job?

To be honest, I felt like a bit of a fraud. I had picked up a Pipetman for the first time in my life just 3 years hence and was now on the job market advertising myself as a researcher capable of running an independent research laboratory! During my last year of clinical training, where I had specialized in hemostasis and thrombosis, I sent out a ton of applications for an assistant professor job in departments of pathology. Thanks to the papers I had published with Bill, I received a number of offers, and decided upon the University of Michigan, Ann Arbor. The chairman, Peter Ward, was most supportive of research and indicated that if I was able to pay for most of my salary from grants, then I could exclusively devote my energies to research. This, and my pediatrician wife getting a clinical training fellowship at Ann Arbor, sealed the deal.

CDD: What were the early years like as an Assistant Professor?

The initial years in Ann Arbor were both exciting and frustrating. I felt the nervous excitement of being an independent researcher. The nervousness stemmed from the fact that there appeared to be an absence of a safety net. I had 5 years to make tenure, during which time I needed to be adequately funded and published—otherwise I’d have to hit the road.

A lot happened in these 5 years. I restarted working on thrombospondin and to my pleasant surprise, the work got amply funded by the NIH. The scientific environment at Michigan grew by leaps and bounds with the recruitment of my colleague and dear friend from St. Louis, John Lowe, with whom I soon started collaborating. Others who were in Ann Arbor or arrived soon thereafter included David Ginsburg, Francis Collins, Jack Dixon, Gary and Betsy Nabel, Craig Thompson, Andrew Feinberg, Gabriel Nunez, Jeffery Leiden, and many other highly driven investigators.

It was during this time that I suffered a crisis of confidence and got frustrated. The work on thrombospondin was proceeding at a rapid pace, but the overall progress was gradual. It reminded me of the acres of posters I had seen at the Cell Biology meeting, each describing a modest advance. I badly wanted to make a larger contribution, do work that would get noticed, and undertake research that would end up in introductory textbooks. There was no question—I had to change fields.

CDD: How did you get involved in cell death research?

During my frustration with the seemingly incremental advances I was making in the study of thrombospondin, I had a chance encounter with Rory Marks, an Australian post-doctoral fellow with Peter Ward, and decided to take a look at the effect of tumor necrosis factor, TNF, on endothelium. This pleiotropic, pro-inflammatory cytokine efficiently converted the anticoagulant surface of endothelium to one that supported coagulation so that blood clotted readily, a happening of importance to many vascular pathologies.

During my initial work and reading, I became fascinated by the fact that certain cancer cells acutely died when exposed to TNF and others could be made sensitive by treating with TNF and cycloheximide, a protein synthesis inhibitor. What was going on here? It seemed that under certain circumstances, the receptor for TNF was able to engage a death pathway! What were its components?

As I was contemplating this, the groups of Peter Krammer in Heidelberg and Shige Nagata at Osaka Biosciences Institute characterized a related receptor, termed Fas or CD95, that under the correct circumstances was a much more potent killer. David Goeddel, the legendary molecular biologist at Genentech and Nagata, showed that the receptors for TNF and Fas, colloquially referred to as death receptors, contained a discrete domain within the cytosolic segment—dubbed the death domain—that was responsible for engaging the enigmatic downstream death pathway. I realized this was an important question, one worthy of attention and if answered, would be of major consequence—as one would be uncovering a new biochemical pathway. This was an opportunity not to be missed!

This was my chance to make a lasting mark! I had a talented MD/PhD student in the laboratory, Muneesh Tewari, Fig. 3, and together we decided to tackle this puzzle. Karen O’Rourke, my technician in St. Louis, had joined me in Ann Arbor and also threw herself into the project. During this time, a major advance had happened in the study of programmed cell death in the worm, *C. elegans*, by the Horowitz laboratory at MIT. They had discovered that the centrally important mediator of death, a protein encoded by the gene Ced-3, was a cysteine protease with an unusual substrate specificity—it cleaved after aspartic acid residues. Indeed, they discerned this only after realizing it had significant sequence homology to the mammalian protease, interleukin-1 converting (ICE) enzyme, that had been cloned and characterized by Nancy Thornberry at Merck and Roy Black at Immunex.

**Fig. 3:**
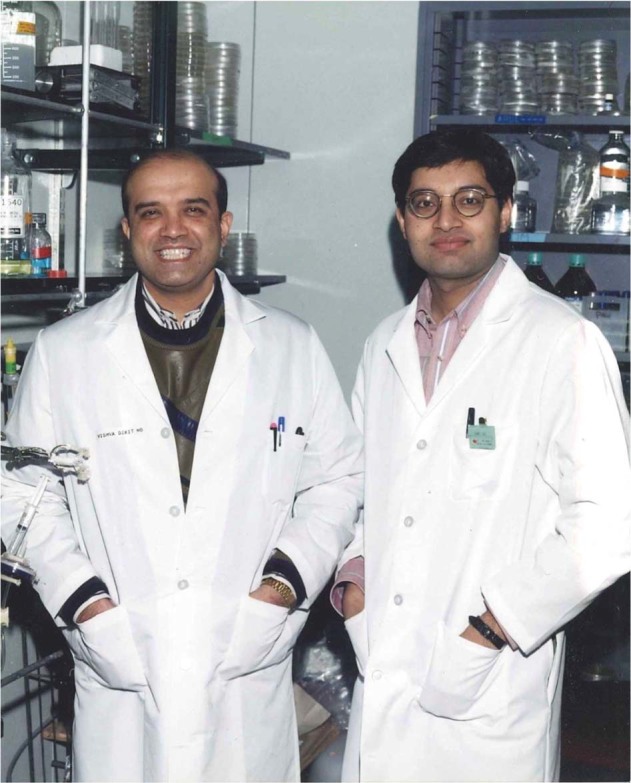
At University of Michigan, Ann Arbor, with Muneesh Tewari

We wondered if ICE or an ICE-like protease was part of our mysterious death pathway in mammalian cells engaged by Fas and TNF-receptor. How could we address this pressing question? Guy SalveCaspasesen, a protease biochemist at Duke, had previously characterized a poxvirus-encoded serpin, CrmA, as a potent inhibitor of ICE. Muneesh made contact and thanks to Guy’s generosity, we soon had a CrmA expression construct in our anxious hands. The experiment was embarrassingly simple: express CrmA and see if TNF and Fas induced death was blocked.

In my office working on a grant late into the night, I heard a commotion coming down the corridor. It was Muneesh! He looked like he had seen Santa Claus! He dragged me to the microscope—CrmA completely blocked cell death [3]. In fact, the cells were now growing happily in the presence of what had previously been a death-inducing stimulus. Just amazing! We were off to the races.

Much has been written about subsequent events and I refer the reader to three personal accounts [4–6]. Suffice it to say that with a group of talented MD/PhD students that included Arul Chinnaiyan, Tony Opipari, and postdoctoral fellows Marta Muzio and James Pan, we defined the essential framework for death-receptor signaling, at times contemporaneously with competing groups. This included molecules that today make up the lexicon of pro-inflammatory and death signaling pathways: A20 (TNFAIP3) [7], FADD [8], caspase-8 (FLICE) [9], caspase-3 (YAMA) [10], caspase-6 (Mch2) [11], caspase-7 (ICE-LAP3) [12], caspase-9 (ICE-LAP6) [13], caspase-10 (FLICE2) [14], caspase-14 [15], TRAF3 (CD40bp) [16], Death-receptor 3 (DR3) [17], Death-Receptor 4 (DR4, TRAIL-R1) [18], Death - Receptor 5 (DR5, TRAIL-R2) [19], Death-Receptor 6 (DR6) [20], Decoy Receptor 2 (TRUNDD) [21], c-FLIP (I-FLICE) [22], v-FLIP [23], RAIDD [24], IRAK2 and MyD88 [25] (recognized in later years -2013- as a “Pillars in Immunology” discovery by the *Journal of Immunology*).

Additionally, working with Guy Salvesen, a number of important concepts were brought to fruition, including (i) the induced proximity model that suggested a mechanism for activation of initiator caspases [26], and (ii) that killing of target cells by cytotoxic T-cells was mediated by granzyme B proteolytically activating caspase-3 [27].

CDD: Tell us about your decision to move to Genentech.

The excitement in those heydays of cell death research was palpable. Each new discovery opened up a whole new vista of research. I was on a roll! In the midst of this excitement, I got a call from a headhunter at Genentech. Would I be interested in a position to be Director of Oncology? Of course not, I thought to myself, but my brother lived in the Bay Area and this would be a chance to visit him, paid for by Genentech! The visit and interview at Genentech, however, went exceptionally well. I was incredibly impressed by the commitment of upper management, including CEO Art Levinson and CMO Sue Hellman, to developing a world-class oncology program. They promised that if I took the job, I could continue to run a research laboratory. This seemed like I could have my cake and eat it too!

Given my background in medicine, I was always interested in drug development as that was such a tangible benefit for patients. I took the plunge and moved to Genentech in the summer of 1997. I inherited and was blessed with a great department that included Napoleone Ferrara, a pioneer in anti-angiogenic therapies; Mark Sliwkowski, who did so much to develop therapeutics targeted to the HER2 axis; Fred de Sauvage, who developed a small molecule inhibitor of the sonic hedgehog pathway; Avi Ashkenazi, a leader in the field of death receptor signaling; and Paul Polakis, who was amongst the first to implicate the Wnt pathway in human cancer. The story of drug development is for another day, but knowing first-hand the trials and tribulations of this byzantine process, in my mind, it is nothing short of a miracle.

CDD: How were you able to continue your basic research at Genentech?

Unusually, Genentech had a spectacular post-doctoral program that was rooted in academia. Fellows were not allowed to work on a product-related project, but rather, had to choose a basic research problem to tackle, ideally one that was imminently publishable. Restarting the laboratory certainly resulted in a loss of momentum, but I was fortunate to attract good post-doctoral fellows and began anew.

Initially, the laboratory started exploring new members of the TNF receptor family and NF-kB signaling in particular. Minhong Yan, Kim Newton, Astrid Ruefli, and Anthony Uren, all talented postdoctoral fellows, soon defined an accessory pathway used by antigen receptors to activate NF-kB. The core complex consisted of three proteins: CARD11, BCL10 and MALT1/paracaspase, that in concert were able to engage the canonical NF-kB pathway [28]. Intriguingly, we discovered that MALT1/paracaspase, a driver of MALT lymphoma, encoded a putative protease—hence we initially termed it paracaspase – as it had features of a cysteine protease containing a caspase fold [29]. Fascinatingly, we discovered related proteases that we termed metacaspases in plants and fungi [29] where they may contribute to the equivalent of programmed cell death, but this needs further substantiation.

Additionally, we became interested in a family of kinases termed RIPKs after RIPK1, a death domain-containing kinase that is part of the proximal TNF receptor signaling complex and plays a role in regulating cell death and NF-kB activation. Initially, we characterized new family members including RIPK2 as an NF-kB activator [30] and RIPK3 as a death inducer [31]. Working with Wayne Fairbrother in Structural Biology, we also discovered two new homotypic interaction motifs: Pyrin and RHIM, both of which would achieve notoriety in later years [32, 33].

During this period, Minhong Yan became interested in defining the receptors for BAFF, a cytokine in the TNF family that is a most potent B-cell mitogen. In short order, using expression cloning, he defined its three receptors: BR3 (BAFF-R), TACI and BCMA. This was a highly competitive affair and we did not emerge as the leader of the pack. This setback made me reassess our work, as I felt we were spread way too thin without a central theme.

Initially, my mind drifted to an intriguing finding by Ingrid Wertz, a newly joined graduate student from the UC Davis Biotechnology Program, and Karen O’Rourke. Looking through the databases for other proteins with sequence similarity to zinc fingers within A20, they stumbled upon RFWD2, also known as COP1, a major inhibitor of plant photomorphogenesis, a process of fundamental importance to plant life and by extension, life on the planet. Why was such a centrally important plant gene conserved in mammalian cells? What was it doing?

Fascinated by this finding, we’ve pursued it over the years. This led to the discovery that COP1 is a substrate binding component of a large multi-subunit Cullin 4A-based E3-ligase that targets key transcription factors including c-Jun, Etv1, Etv4, and Etv5 for proteosomal degradation [34]. Inspired by this project, we invested considerable effort in characterizing ubiquitin hydrolases that counter the activity of E3-ligases as regulators of cellular signaling—in particular, BAP1, a histone modifier that is mutated in a highly penetrant form of familial cancers [35].

CDD: Tell us about your present department: Physiological Chemistry.

After 10 years of running the Oncology Department, I decided it was time to do something else—to go back to my interest in basic discovery and devote my energies primarily to that enterprise. Genentech was most understanding and allowed me to form a small department: Physiological Chemistry, and I hired two former postdoctoral fellows, Kim Newton and Nobuhiko Kayagaki, as scientists, with the understanding that we would join forces and undertake a collaborative venture to unravel the complex interplay between cell death and inflammation at the molecular level. It has been, and continues to be, a marriage made in heaven as we’ve worked as a cohesive collaborative enterprise with much success and a sense of fulfillment, really enjoying and appreciating what we do.

CDD: How did this interest in Cell death driving Inflammation arise?

This was rekindled when a postdoctoral fellow, Sanjeev Mariathasan, who was attempting to define the physiological role of the inflammasome—a molecular apparatus responsible for inflammatory cell death—made an unexpected discovery. The approach was generating appropriate knockout mice of putative inflammasome components and confirming their involvement in vivo. He initially focused on the adaptor ASC and showed that the KO was unresponsive to all activators of the inflammasome, suggesting it was part of a central conduit. The paper describing the result was rejected by *Nature* with the criticism that the findings were predictable.

In response, we thought it best to run a negative control to show that not all purported inflammasome components defined by in vitro experimentation had an in vivo phenotype. Kim Newton had knocked out NLRC4 (IPAF1), a relatively obscure component of the inflammasome, and we decided this would be our negative control, as null mice lived to a ripe old age without any untoward effect. Much to our surprise, it responded to inflammasome activators including ATP and nigericin, but failed to respond to *Salmonella*. It was as if NLRC4 null macrophages were totally oblivious to the presence of this intracellular pathogen! Since this was the first evidence that the inflammasome possessed components that only responded to specific stimuli, the revised manuscript was readily accepted and was met with much interest by the community [36].

Subsequently, with the identification of NLRP3 as a specific sensor for ATP and nigericin, by ourselves and others, it became evident that the inflammasome possesses specificity as dictated by sensors that only respond to specific insults. Mohamed Lamkanfi, an ambitious postdoctoral fellow from Belgium, joined us to continue work on the NLRP3 inflammasome and to everyone’s surprise, showed that it was a druggable target! He found that analogues of the hypoglycemic drug, Glyburide, were capable of inhibiting this pathway [37] and today a number of NLRP3 inhibitors are headed to the clinic.

CDD: What does the Department work on now?

Kim’s group has largely devoted itself to the study of components of the necroptotic pathway and the complicated interplay between these components has only been possible to dissect using complex genetically engineered mouse models [38, 39]. Hence much time and effort are spent on generating them. These, however, have been invaluable in her collaboration with Domagoj Vucic—also a former postdoctoral fellow—who now leads the research program to develop a therapeutic RIPK1 inhibitor.

Nobuhiko’s group discovered the non-canonical inflammasome pathway that responds to the presence of intracellular LPS independent of toll-like receptors [40–42]. This pathway has received much attention of late as it mediates gram-negative endotoxic shock, and its two central components, caspase-4 and Gasdermin D, are attractive drug targets. Much effort is being expanded by his group to uncover additional components in this pathway.
